# Factors associated with obstetric fistula among reproductive age women in Ethiopia: a community based case control study

**DOI:** 10.1186/s12978-023-01622-y

**Published:** 2023-05-23

**Authors:** Ataklti Gebretsadik Woldegebriel, Gebremedhin Gebreegziabiher Gebrehiwot, Abraham Aregay Desta, Kiros Fenta Ajemu, Asfawosen Aregay Berhe, Tewolde Wubayehu Woldearegay, Kiros Demoz Ghebremedhin, Nega Mamo Bezabih

**Affiliations:** 1 Tigray Health Research Institute, Mekelle, Tigray Ethiopia; 2 Adigrat University, Adigrat, Tigray Ethiopia

**Keywords:** Obstetric fistula, Maternal morbidity, Ethiopia

## Abstract

**Background:**

Obstetric fistula is a major public health concerns in Ethiopia. It is the most devastating cause of all maternal morbidities.

**Method:**

Data from the 2016 Ethiopian Demographic Health Survey (EDHS) was analyzed. A community-based unmatched case control study was conducted. Seventy cases and 210 non cases were selected using random number table. Data were analyzed by using STATA statistical software version 14. Multivariable logistic regression model was applied to determine the factors associated with fistula.

**Results:**

The majority of fistula cases were from rural residences. The multivariable statistical model showed that rural residence (Adjusted OR (AOR) = 5, 95% CI 4.26, 7.52), age at first marriage (AOR = 3.3, 95% CI 2.83, 4.60), poorest wealth index (AOR = 3.3, 95% CI 2.24, 5.01) and decision making for contraceptive use by husband alone (AOR = 1.3, 95% CI 1.124, 1.67) were factors significantly associated with obstetric fistula.

**Conclusion:**

Age at first marriage, rural residence, poorest wealth index and decision making for contraceptive use by husband alone were significantly associated factors for obstetric fistula. Intervening on these factors will reduce the magnitude of obstetrics fistula. In this context there is in-need to improve on avoiding early marriage through awareness creation to the community and developing legal framework by the policymakers. Furthermore, information about the joint decision making to use contraceptives should be disseminated though mass-media and interpersonal channels.

## Plain language summary

Globally, 2–3 million women are affected by obstetric fistula. It is common in developing nations, mainly in sub-Saharan Africa and Southeast Asia where suitable, timely care and treatment are hard to find. This devastating condition adversely affects women’s physical and mental health. There is little research conducted in relation to the experiences of women living with leakage of urine and/or feces in Ethiopia. Therefore, this study provides a better understanding on obstetric fistula and its associated factors.

The present study used the recent Ethiopia Demographic Health Survey (EDHS) 2016, to determine the associated factors of obstetric fistula among reproductive age group women (15–49 years) in Ethiopia.

In this study rural residence, poorest wealth index, age at first marriage less than 18 years, and decision making for contraceptive use by husband alone were the associated factors of obstetric fistula. Intervening on these factors will reduce the magnitude of obstetrics fistula. In this context there is in-need to improve on avoiding early marriage through awareness creation to the community and developing legal framework by the policymakers. Furthermore, information about the joint decision making to use contraceptives should be disseminated though mass-media and interpersonal channels. Comprehensive intervention strategies should be customized at different government hierarchies to combat and reduce obstetric fistula.

## Background

Obstetric fistula is an atypical link between the vagina, rectum, and/or bladder that may arise after protracted and obstructed labor. Among all maternal morbidities; obstetric fistula is an extreme and debilitating hardship of childbirth and maternal morbidity [1].Globally, each year between 50 000 to 100 000 women are affected by obstetric fistula [2].

Fistula remains a major public health challenge in low-income countries, mainly in sub-Saharan Africa and Southeast Asia where suitable and timely obstetrical care is hard to find. It is estimated that more than two million young women survive with untreated obstetric fistula in Asia and sub-Saharan Africa [3].

Ethiopia’s fertility rate is among the uppermost countries in the world and almost half (48%) of births attend by a skilled birth attendant. Besides, rural areas where 80% of the population resides, poor and under-nourished women countenance superior risk and challenges to obstetric fistula reduction [4].

Women affected by obstetric fistula are often abandoned by their husbands and stigmatized by the community. Hence, leads to low self-esteem, depression and long lasting emotional trauma [5]. According the USAID report, it is estimated that nine thousand women in Ethiopia develop obstetric fistula every year, and that up to hundred thousand women are living with untreated fistula [6].

Available evidences have shown that, major risk factors for obstetrics fistula include early marriage, rural place of residence, poverty, illiteracy, duration of labor, respondent height, and lack of emergence obstetric care [7–11].

**Fig. 1:**
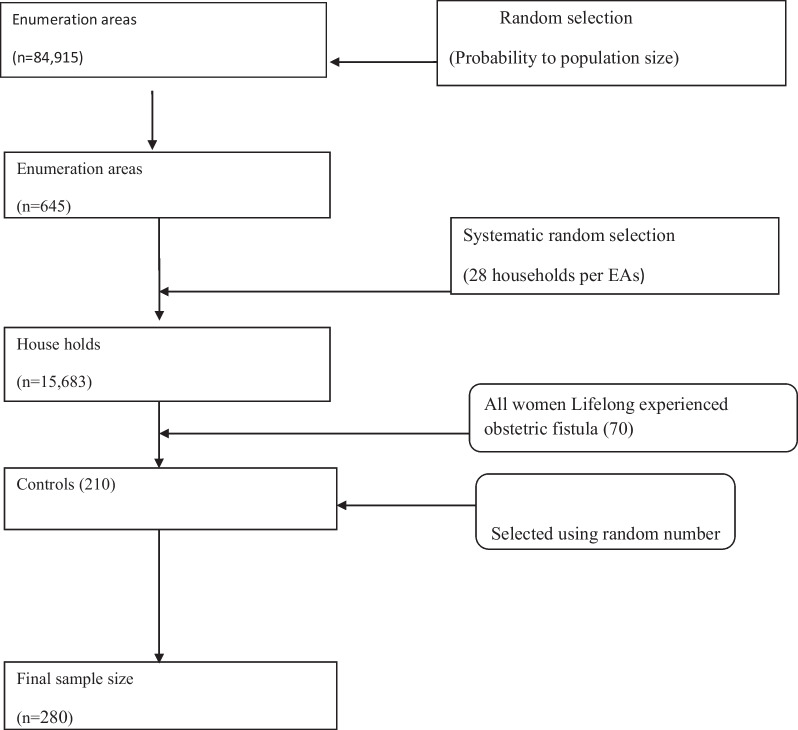
Flow chart of sample selection technique EDHS 2016 (n = 280)

**Fig. 2:**
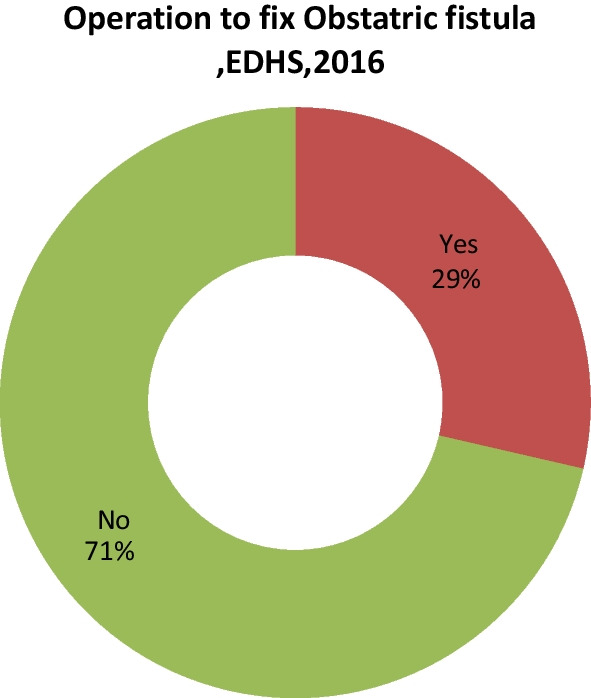
Number of operation to fix obstetric fistula cases, EDHS (n = 70)

Community based surveys generally provide wider coverage, better representation of national population and more opportunities to collect a wide range of data. Due to nationally representative samples and use of similar questions across surveys, the EDHS surveys provide a unique set of data to assess factors associated with obstetric fistula. Identifying factors associated with obstetric fistula using various study design is mandatory (Figs. 1 and 2).

To our knowledge, no nationally representative community based study with case control study design has documented to identify risk factors for obstetric fistula in Ethiopia. Therefore, this study was aimed to identify the risk factors for obstetric fistula from a local context among women in Ethiopia.

## Methods

### Data source and sampling techniques

We used the dataset of women aged 15–49 years included in the EDHS 2016. Ethiopia is sub divided in to eleven geographical regions which are sub divided into kebeles (the smallest administrative unit). Each region was stratified in to urban and rural areas. The 2016 EDHS sample is stratified and was selected in two stages.

In the first stage, 645 enumeration areas were randomly selected: 202 in urban areas and 443 in rural areas. In the second stage, a fixed number of 28 households per cluster were selected randomly for each enumeration areas. The 18,060 households were randomly selected and 16,650 households were eligible and interviewed. Additional information about the methodology of EDHS 2016 can be accessed in the published report of the main findings of the survey [12].

The main focus of this study was women aged 15–49 years, from the EDHS 2016 dataset with various socioeconomic, obstetric and nutrition variables.

### Sample design

In-depth secondary analysis was conducted; the data was obtained from 2016 EDHS, which was taken from Central Statistical Agency (CSA). It is the fourth survey conducted in Ethiopia, following the 2011. A community-based unmatched case–control study was conducted among reproductive age women to identify the factors associated with obstetric fistula.

### Variables and measurements:

#### Dependent variable

The main outcome variable was obstetric fistula, which is defined as reproductive aged women experiencing lifelong obstetric fistula.

#### Independent variables

The selection of the independent variables was guided by the literature and availability of the variables in the dataset. The variables were categorized in to five groups: Maternal, Household, Obstetric, Anthropometric and wealth indices factors.

#### Maternal factors

Maternal age, maternal educational status, maternal antenatal care follow up, marital status, mother’s current employment status.

#### Household factors

number of household members, residence, wealth indices ranked in to five categories (poorest, poorer, middle, richer and richest), sex of household head.

#### Obstetric factors

Place of delivery**,** ANC follow up, size of child at birth, postnatal checkup, Preceding birth interval, Height (Cm) and ever had a terminated pregnancy.

#### Anthropometric measurements

Anthropometric data were collected through measurement of height and weight among all reproductive age women. Among four possible anthropometric indicators to evaluate women’s chronic under nutrition height less than 145 cm; body mass index (BMI) < 18.5 (thinness); weight less than 45 kg; and mid-arm circumference (MUAC) < 22.5 cm. BMI is defined as weight in kilograms divided by height squared in meters (kg/m^2^) [13].

### Wealth index

A wealth index in the EDHS survey was measured based on household asset and data to classify individuals into 5 wealth indices (poorest, poorer, medium, richer and richest). Variables incorporated in the wealth index were ownership of chosen household assets (television, bicycle or car), size of agricultural land, number of livestock and materials used for house construction [13, 14].

### Data analysis

Data analysis was carried out using STATA version 14 (Stata Corp, College station, Texas United states). The data explore for inconsistency and missing value. In all analysis, sample weights have done due to two stage cluster sampling design in the EDHS dataset to adjust for the imbalance probability selection among the strata [12]. Categorical type of data was analyzed by descriptive statistics (frequency and percentage).

Logistic regression analysis was used to identify factors associated with obstetric fistula. Bivariable analysis was carried out to see the crude association of each independent variable with the outcome variable (Obstetric fistula). Those independent variables with *P*-value ≤ 0.05 in the bivariable analysis were included in the final multivariable logistic regression analysis to adjust for confounders and to identify the final factors associated with obstetric fistula.

Logistic regression enter method was used during the multivariate logistic regression analysis. Before inclusion of predictors to the final logistic regression model, the multi-collinearity was checked using VIF < 10/Tolerance > 0.1 for continuous independent variables. The goodness of fit of the final logistic model was tested using Hosmer and lemeshow test at p value of > 0.05. The strength of association of the predictors and outcome variables has been indicated by adjusted odds ratio at 95% confidence interval. The significant association was declared at p ≤ 0.05 for the final logistic regression model.

### Ethical considerations

The study permission was obtained from measure DHS project website to access the dataset (http://www.measuredhs.com)**.**

## Results

### Socio-demographic and other characteristics for cases and controls of the mothers

From a total of 280 samples size, (70 cases and 210 controls) were included in the final analysis. Majority of the cases were aged 34–49 (44%) while controls were aged 15–23 years 37.1%. More than half (57.1%) of the mothers in cases and 42.4% the controls had no education. Majority of cases (84.3%) and 64.8% controls were living in rural residence.

Regarding, wealth index 30% of mothers in cases and 24.3% in controls were poorest. More than (62%) of cases and 63.3% non controls have equal or greater than four household members. The majority (68.6% and 58.6%) of mothers in cases and control group were married respectively. More than half (52.9%) of mothers in cases and 43.3% in controls were Orthodox followers. Above one third of mothers in cases and 46.7% in non-controls had had a work (Table 1).

**Table 1:** Socio-demographic and other characteristics for cases and controls of the mothers, EDHS, 2016

Variables	Category	Cases (n = 70) Freq (%)	Controls (n = 210) Freq (%)
Maternal current age (years)	15–23	16 (22.9)	78 (37.1)
	24–33	23 (32.9)	67 (31.9)
	34–49	31 (44.3)	65 (31)
Maternal educational status	No education	40 (57.1)	89 (42.4)
	Primary	22 (31.4)	74 (35.2)
	Secondary	6 (8.6)	32 (15.2)
	Higher	2 (2.9)	15 (7.1)
Residence	Urban	11 (15.7)	74 (35.2)
	Rural	59 (84.3)	136 (64.8)
Wealth index			
	Poorest	21 (30.0)	51 (24.3)
	Poorer	10 (14.3)	26 (12.4)
	Middle	14 (20.0)	26 (12.4)
	Richer	10 (14.3)	27 (12.9)
	Richest	15 (21.4)	80 (38.1)
Number of house hold members			
	1	36 (12.9)	29 (13.8)
	2–4	70 (25.0)	48 (22.9)
	> 4	174 (62.1)	133 (63.3)
Maternal occupation			
	No	27 (38.6)	98 (46.7)
	Yes	43 (61.4)	112 (53.3)
Marital status			
	Never in union	6 (8.6)	56 (26.7)
	Married	48 (68.6)	123 (58.6)
	Living with partner	2 (2.9)	4 (1.9)
	Widowed	4 (5.7)	9 (4.3)
	Divorced	8 (11.4)	13 (6.2)
	No longer living together/separated	2 (2.9)	5 (2.4)
Religion/Religious			
	Orthodox and Catholic	37 (52.9)	92 (43.8)
	Protestant	7 (10.0)	49 (23.3)
	Muslin and others	26 (37.1)	69 (32.9)

### Obstetric characteristics of cases and controls

More than half (58.6%) of cases and 62.9% of controls gave birth at home. Twenty eight percent of the cases and 24.7% in controls were not followed Antenatal care.

About 18% and 21.6% of cases gave birth to very large and larger than average babies. Whereas, about 16% and 18.9% of controls gave birth to very large and larger than average babies.

.Majority of mothers (96.6%) of cases and 86.6% of controls were not followed postnatal checkup. Greater than one fourth number of mothers (31.3%) of cases and 23.6% of controls were with less than 24 months birth interval. Almost majority of the cases (85.7%) and 88.1% of controls were taller than 150 cm. Majority of cases (80%) and 90.5% of controls had never terminated pregnancy (Table 2).

**Table 2:** Obstetrics characteristics of cases and controls of the mothers, EDHS, 2016 (n = 280)

Variables	Category	Cases (n = 70) Freq (%)	Controls (n = 210) Freq (%)
Place of delivery	Home	17 (58.6)	61 (62.9)
	Health facilities	12 (41.4)	36 (37.1)
ANC follow up	No ANC visit	8 (27.6)	24 (24.7)
	One	1 (3.4)	8 (8.2)
	Two	1 (3.4)	8 (8.2)
	Equal or greater Three	19 (65.5)	57 (58.8)
Size of child at birth			
	Very large	9 (18)	8 (21.6)
	Larger than average	8 (16)	7 (18.9)
	Average	20 (40)	12 (32.4)
	Smaller than average	5 (10)	5 (13.5)
	Very small	7 (14)	5 (13.5)
	Don't know	1 (2.0)	0 (0)
Postnatal check up			
	No	28 (96.6)	84 (86.6)
	Yes	1 (3.4)	13 (13.4)
Height (Cm)			
	Less than 150	10 (14.3%)	25 (11.9)
	Equal or greater than 150	60 (85.7)	185 (88.1)
Preceding birth interval (months)			
	< 24	15 (31.3)	25 (23.6)
	Equal or greater than 24	33 (68.8)	81 (76.4)
Ever had a terminated pregnancy			
	No	56 (80.0)	190 (90.5)
	Yes	14 (20.0)	20 (9.5)

### Factors associated with obstetric fistula among mothers aged 15– 49 years

In bivariable logistic regression analysis, age at first marriage, height, wealth index place of residence, owning television, level of literacy, number of household members, ever had a terminated pregnancy and decision making in using contraceptive by husband alone were significantly associated with obstetric fistula. However, in multivariable logistic regression analysis: age at first marriage, poorest wealth index, rural residence and decision making for contraceptive use by husband alone were significantly associated variables with obstetric fistula.

In the multivariable logistic regression analysis age at first marriage with less than 18 years were 3.3 times (AOR = 3.39; 95% CI: 2.832, 4.601) more likely higher in developing fistula compared to age at first marriage greater than 18 years old. Wealth index poorest category were 4.6 times (AOR = 4.62; 95% CI: 2, 238, 5.015) more likely higher in developing fistula compared to the richest wealth index. Rural residents were 5.14 times (AOR = 4.62; 95% CI: 4.262, 7.521) more likely higher developing fistula compared to urban residents. Decision making for contraceptive use by husband alone were 1.3 times (AOR = 4.62; 95% CI: 1.124, 1.670) more likely higher in developing fistula compared to joint decision making (Table 3).

**Table 3:** Factors associated with anemia using bivariate and multivariable logistic regression model (n = 280)

Variables		Experienced Obstetric Fistula		COR (95% CI)	AOR (95% CI)
		Yes n (%)	No n (%)		
Age at first marriage (Years)	< 18	62 (88.6)	163 (77.6)	3.717 (3.123,5.671)	3.294 (2.832,4.601)*
	≥ 18	8 (11.4)	47 (22.4)	1	
Height (Cm)	< 150	10 (14.3)	25 (11.9)	1.982 (1.321,2.211)	1.391 (0.823,1.932)
	> 150	60 (85.7)	185 (88.1)	1	
Wealth index	Poorest	21 (30)	51 (24.3)	4.621 (2.998,6.123)	3.34 (2,238,5.015)*
	Poorer	10 (14.3)	26 (12.4)	0.871 (0.745,1.017	0.79 (0.910, 1.014)
	Middle	14 (20.0)	26 (12.4)	1.037 (0.881,1.221)	1.0124 (0.761,1.011)
	Richer	10 (14.3)	27 (12.9)	1.124 (0.889,1.421)	
	Richest	15 (21.4)	80 (38.1)	1	
Residence	Urban	11 (15.7)	74 (35.2)	1	
	Rural	59 (84.3)	136 (64.8)	6.123 (4.981,8.413)	5.141 (4.262,7.521)*
Has television	No	61 (87.1)	150 (71.4%)	2.323 (1.721,2.986)	1.982 (0.788,1.534)
	Yes	9 (12.9)	60 (28.6)	1	
Literacy	Literate	24 (34.3)	102 (48.6)	0.934 (0.623,1.943)	0.711 (0.591,1.544)
	Illiterate	46 (65.7)	108 (51.4%)	0.763 (0.589,2.343)	0.612 (0.582,1.923)
Number of house hold members					
	1	7 (10.0)	29 (13.8)	1	
	2–4	22 (31.4)	48 (22.9)	1.258 (0.890,1.395)	0.726 (0.561,1.133)
	> 4	41 (58.6%)	133 (63.3)	2.912 (1.631,3.211)	1.942 (0.823,1.232)
Ever had a terminated pregnancy	No	56 (80)	190 (90.5%)	2.341 (0.672,1.675)	
	Yes	14 (20)	20 (9.5)	1.971 (0.925,1.321)	
Women’s Current Contraceptive Method Decision Making	Mainly respondent	3 (4.8)	12 (25)	1.258 (0.994,1.592)	
	Mainly husband, partner	16 (25)	3 (6.3)	1.554 (1.248,1.693)	1.308 (1.124,1.670)*
	Joint decision	44 (69.3)	33 (68.8)	1	

Remark: *P < 0.05, **P < 0.001, ***P < 0.0001,

## Discussion

This study was aimed to identify the risk factors for obstetric fistula from a local context among women in Ethiopia. This study was a case control study, which analyzed risk factors for obstetric fistula among reproductive age women in Ethiopia from the 2016 EDHS dataset.

This study analysis identified that age at first marriage, rural residence, poorest wealth index and decision making to use contraceptive by husband alone were significantly associated with obstetric fistula. Age at first marriage was significantly associated with obstetric fistula. This result in lines with the study findings conducted in developing countries in Tigray, Ethiopia [7], Sub-Saharan Africa [15], and Uganda [16].

The possible justifications for this is might be due to experiencing of obstetric fistula with early age at first marriage in young adolescents before the pelvic girdle is fully developed and elevated risk of distress from obstetric fistula while giving birth.. Living in rural place is the major risk factor for obstetric fistula, as evidenced by studies conducted, in Eretria [17] and Democratic Republic of Congo [18].

This difference might be due to lack of access to maternity health service lack of awareness of institutional obstetric service poor economic level to travel to get health care service..

In this study, attempt was made to demonstrate the association of obstetric fistula with wealth index, being poorest wealth index has been shown more likely developing obstetric fistula than the richest wealth index. This findings in line with the studies in South-eastern India [19], Tanzania [20], Uganda, [21] and Nairobi, Kenya [22]. This might be due to women who had a better wealth index might afford to pay and transport to the health care services..

This research finding indicated that decision making for contraceptive use by husband alone was more likely in developing fistula compared to the ioint decision making. This is consistent with previous studies conducted in sub-Saharan Africa [23] and North Western, Nigeria [24]. The possible reason might be that the women for non-use of modern contraceptive methods were husband antagonism, desire for additional children, and religious ban. This is not amazing as culture and religion has positioned men as the top of the family and women cannot make decisions concerning health care service.

The findings of this study will bring much needed attention to this serious condition and provide information to help those who are most likely to develop an obstetric fistula. Further longitudinal studies are needed to determine other possible potential factors.

## Strengths and limitations of the study

The strength of this study was; the analysis used a national representative data which permits to generalize to the population. It also functionalizes all the DHS data principles like weighting. The limitation of this study was its cross-sectional data collection, which cannot examine causation of the precedence in time between exposure and outcome. There were some missing values for some variables in the dataset. Being; the cases for obstetric fistula were lifelong therefore, the authors might fail to consider some factors which could affect the interpretation of the results, maternal verbal reports and recall bias might have been introduced.

## Conclusions

Age at first marriage, rural residence, poorest wealth index and decision making for contraceptive use by husband alone were significantly associated factors for obstetric fistula. Intervening on these factors will reduce the magnitude of obstetrics fistula. In this context there is in-need to improve on avoiding early marriage through awareness creation to the community and developing legal framework by the policymakers. Furthermore, information about the joint decision making to use contraceptives should be disseminated though mass-media and interpersonal channels.

## Data Availability

All data supporting the findings of this study are available at http://www.measuredhs.com

## Abbreviations

ANC: Antenatal care
BMI: Body mass index
DHS: Demographic Health Survey
EDHS: Ethiopia Demographic Health Survey
OF: Obstetric fistula
IF: Variance Inflation Factor
USAID: United States Agency for International Development
